# Molecular dynamics simulations reveal how vinculin refolds partially unfolded talin rod helices to stabilize them against mechanical force

**DOI:** 10.1371/journal.pcbi.1012341

**Published:** 2024-08-07

**Authors:** Vasyl V. Mykuliak, Rolle Rahikainen, Neil J. Ball, Giovanni Bussi, Benjamin T. Goult, Vesa P. Hytönen

**Affiliations:** 1 Faculty of Medicine and Health Technology, Tampere University, Tampere, Finland; 2 School of Biosciences, University of Kent, Canterbury, United Kingdom; 3 Scuola Internazionale Superiore di Studi Avanzati, SISSA, Trieste, Italy; 4 Fimlab Laboratories, Tampere, Finland; University of Virginia, UNITED STATES OF AMERICA

## Abstract

Vinculin binds to specific sites of mechanically unfolded talin rod domains to reinforce the coupling of the cell’s exterior to its force generation machinery. Force-dependent vinculin–talin complexation and dissociation was previously observed as contraction or extension of the unfolded talin domains respectively using magnetic tweezers. However, the structural mechanism underlying vinculin recognition of unfolded vinculin binding sites (VBSs) in talin remains unknown. Using molecular dynamics simulations, we demonstrate that a VBS dynamically refolds under force, and that vinculin can recognize and bind to partially unfolded VBS states. Vinculin binding enables refolding of the mechanically strained VBS and stabilizes its folded α-helical conformation, providing resistance against mechanical stress. Together, these results provide an understanding of a recognition mechanism of proteins unfolded by force and insight into the initial moments of how vinculin binds unfolded talin rod domains during the assembly of this mechanosensing meshwork.

## Author summary

Cells attach to and interact with their surroundings, and sense mechanical signals from the microenvironment. Vinculin and talin are classical examples of mechanosensitive proteins that complex in a force-dependent manner. Talin domains unfold under mechanical loads exposing buried vinculin binding sites, which are single α-helices. Such mechanical stretching enables recognition by vinculin, however, the structural states of vinculin’s binding site when under force and the complexation mechanisms are poorly understood. We used molecular dynamics simulations to study the behavior of a single vinculin binding helix under mechanical load and during the initial moments of the vinculin–talin binding process. Our results reveal the highly dynamic nature of a vinculin binding helix under mechanical load. Depending on the force magnitude, the helix frequently reforms to a partially folded conformation on the nanosecond to microsecond timescale. Crucially, vinculin recognizes such partially folded helices in talin and promotes their refolding against force. Our findings are beyond the resolution limits imposed by experimental single molecule methods, suggesting a model which may be useful as a general mechanism for protein-protein binding under mechanical load.

## Introduction

**Fig 1:**
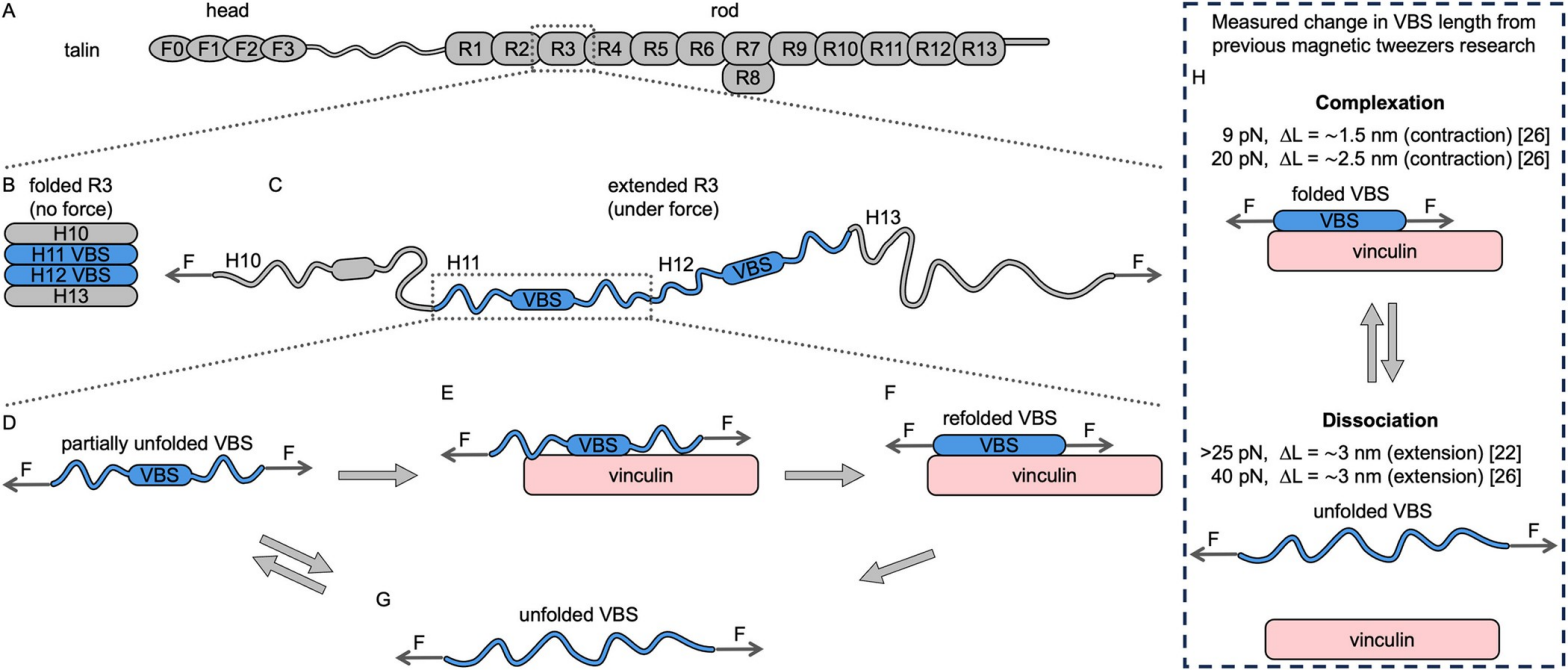
Schematic representation of talin unfolding under force and VBS refolding upon vinculin binding. Structure of (A) full talin in folded conformation, and rod R3 domain in both (B) folded and (C) extended conformation under force. (D) Partially unfolded VBS under force either (E) binding vinculin and (F) refolding, or (G) undergoing further unfolding. (H) Summary of magnetic tweezers experiments where vinculin–talin binding and dissociation under force have been observed; as contraction [26] and extension [22,26] in talin end-to-end distance.

Cells attach to the extracellular matrix (ECM), sense its elasticity, and respond to these mechanical signals, converting them into biochemical ones [1–3]. Focal adhesions are macromolecular complexes where transmembrane integrin receptors bind the ECM and couple it to the actin cytoskeleton via adapter proteins such as talin and vinculin [4–7]. Recruitment of vinculin to focal adhesions is force-regulated [8–10]. Talin binds to the cytoplasmic tail of the integrin receptor via its N-terminal head domain and to actin via its large C-terminal rod domain, which consists of 13 bundles, each formed by 4 or 5 α-helices (Fig 1A) [11]. Nine of the 13 talin rod domains contain 11 cryptic vinculin binding sites (VBSs) [12]. The vinculin head domain 1 (VD1) binds these VBS helices in talin and the vinculin tail domain binds to the actin cytoskeleton, further reinforcing focal adhesions [13]. Binding of vinculin to talin involves embedding the VBS into the VD1 structure as a fifth helix – the helix-addition mode of binding [14], where hydrophobic interactions are critical for initial recognition and drive the complexation [15,16]. The talin rod domains require mechanical stretching to expose these buried VBSs [17,18] by complete or partial unfolding [19,20], and thus vinculin recruitment depends on the mechanical loading on talin (Fig 1) [21–23]. Talin rod domains differ in their mechanical stability and can be unfolded at forces up to 25 pN [24,25]. R3 is known to be one of the mechanically weakest rod domains, unfolding at ∼5 pN force [22,25]. R3 is a 4-helix bundle and has two VBSs, H11 and H12, providing the initial site for recruiting vinculin upon exposing its VBSs at low forces.

Previous research shows that vinculin and talin dissociate at high forces (above 25 pN), observed as an increase in talin end-to-end distance of ∼3 nm, which is attributed to unfolding of the VBS helix to a random coil [22,26]. In contrast, vinculin binding to talin R3 results in a reduction in talin end-to-end distance under tension (~1.5 nm at 9 pN and ~2.5 nm at 20 pN), suggesting that VBS helices undergo a coil-to-helix transition upon vinculin recognition and binding [26] (Fig 1H). However, how the complexation occurs when the VBS structure is destabilized by force remains unclear. Furthermore, it is unknown what VBS conformations permit recognition by vinculin. Understanding the recognition mechanism of proteins unfolded by force is a central question in mechanobiology.

To learn how mechanically-tensioned VBS helices can be recognized by vinculin, we used molecular dynamics (MD) simulations with parallel supercomputing. The selected MD approach enabled us to recapitulate the complex protein structural changes that can be measured experimentally. However, crucially, MD simulations also enabled us to identify new features that are invisible experimentally. Our results demonstrate that upon mechanical unfolding of a talin rod domain, a VBS exhibits fast partial refolding under mechanical load. We find that vinculin can recognize partially unfolded VBS. Upon recognition, vinculin strongly stabilizes the VBS helical form against extension by force which facilitates its refolding, enabling the formation of the high affinity complex between vinculin and the fully folded VBS helix.

## Results

In a folded helical bundle within the talin rod, each VBS exists as a constitutive helix. However, once the bundle has unfolded, the helical form is just one possible state of the VBS, and in isolation, helices are usually thought to exhibit only helical propensity. To determine the structural conformations of free VBS that exist when a domain of talin rod is mechanically unfolded, we used a combination of force-free equilibrium MD and steered MD (SMD) simulations with mechanical load applied to the termini of the VBS. As R3 is the first talin rod domain to unfold, we focused on the two VBSs in R3, helices 11 and 12 of the talin rod, hereafter referred to as H11 and H12.

### Free VBS partially refolds under force on the nanosecond to microsecond timescale

**Fig 2:**
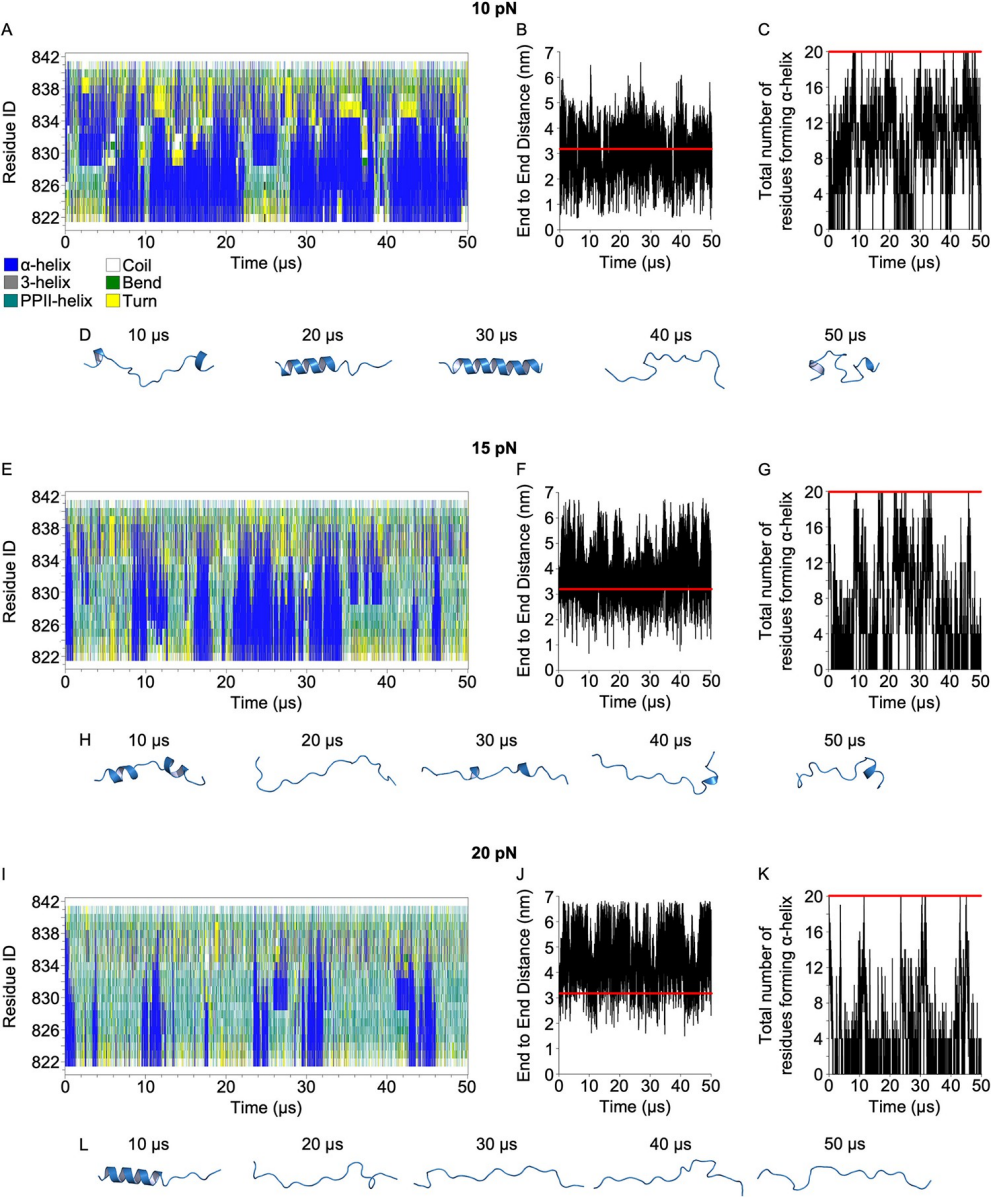
VBS dynamically unfold and reform under loading. (A,E,I) Secondary structure evolution, (B,F,J) end-to-end distance, (C,G,K) number of residues in α-helical conformation, and (D,H,L) intermediate snapshots for H11 VBS from R3 under force at (A-D) 10 pN, (E-H) 15 pN, and (I-L) 20 pN, applied to the termini of VBS. Red line in panels B,F,J indicates the length of the fully folded VBS helix, which is approx. 3.2 nm. Red line in panels C,G,K indicates the number of residues forming α-helix, when the VBS is fully folded. This is equal to 20 residues in the 22-residue VBS construct used.

In force-free simulations, both H11 and H12 showed significant fluctuations of the helical structure and end-to-end distance. The α-helical structure was unstable and partial unfolding was observed (S1 Fig). In SMD, we used different force regimes (10–20 pN) as cellular components are known to experience this force range, and individual talin domains have been observed to unfold within this force magnitude [19,22,24–26]. First, we conducted 1 μs simulations and both VBSs showed dynamic unfolding, adopting an extended conformation (S2 Fig). To reveal VBS conformation after reaching complete unfolding under these physiological forces, we conducted longer simulations using H11 (Fig 2). Constant force at 10 pN did not lead to great extension of end-to-end distance (average (AV) = 3.19 nm, standard deviation (STD) = 0.82 nm) and allowed significant dynamic fluctuations of the VBS conformation, including bending into a hairpin state. The VBS showed highly dynamic structural states but was found to contain α-helicity (at least 4 residues in α-helical conformation) for 92% of the simulation time. The total amount of α-helical content in the 50 μs long trajectory was 55%. Increasing the force to 15 pN led to the VBS being more extended (AV = 4 nm, STD = 0.79 nm) and the helix was refolding less frequently and less significantly, forming α-helicity for 73% of the simulation time. The total amount of α-helical content was 36%. At 20 pN, the VBS conformation adopted an even more extended conformation (AV = 4.73 nm, STD = 0.81 nm). Strikingly, even at 20 pN force, the VBS was refolding frequently, containing an α-helical conformation of at least 4 residues for 49% of the simulation time. The total amount of α-helical content in the SMD trajectory was 20%. These findings indicate that VBS helix has highly dynamic structure and is able to refold significantly even under relatively high mechanical load.

In agreement with previous findings, lower forces permit the formation of compact autoinhibited VBS conformations in our simulations, including hairpin-like states [27]. We selected a threshold for the end-to-end distance of 3 nm, considering that VBS is in an autoinhibited compact state when the length is below 3 nm. Under 10 pN, the VBS end-to-end distance was 3 nm or greater 65% of the simulation time, while at 15 pN 90%, and at 20 pN 97%, in line with previous studies, which suggested that modest mechanical force applied on VBS makes it more accessible for vinculin binding [27].

To quantify the existence of partially refolded states when the VBS is extended and available for recognition by vinculin, we calculated the existence of uninterrupted α-helical structure within the VBS peptide when the end-to-end distance is 3 nm or greater (Table 1). The analysis demonstrates that while refolding to fully folded VBS is rare, partially reformed α-helix states are populated under these forces.

**Table 1:** Percentage of simulation time when VBS contains uninterrupted α-helical conformation with the end-to-end distance of 3 nm or greater. The first microsecond was omitted from the analysis to allow initial unfolding of the starting folded conformation.

Number of residues forming continuous α-helix	Force magnitude		
	10 pN	15 pN	20 pN
≥ 4	59%	64%	47%
≥ 8	43%	34%	21%
≥ 12	27%	20%	10%
≥ 16	10%	8%	3%
20 (fully folded)	2%	1%	0.5%

### Vinculin-bound VBS withstands high mechanical loading

Next, we studied how vinculin binding would influence the stability of the VBS. First, we conducted force-free equilibration of vinculin-bound VBS. In contrast to the highly dynamic conformation observed for isolated helices (Figs 2 and S1), both vinculin-bound H11 and H12 were highly stable, and we did not observe any significant fluctuations of the VBS conformation (S3 Fig). This confirms that binding of vinculin strongly stabilizes the VBS in an α-helical conformation.

To quantify the stabilizing contribution of vinculin binding, we performed comparative analysis of unfolding of isolated VBS helices and vinculin–VBS complexes using constant velocity pulling SMD. The pulling force was applied to the C-alpha atoms of the N- and C-terminal residues of the VBS to unfold it by extending the termini. To get closer to pulling velocities used in experimental setups, we employed low pulling speed (0.01 nm/ns) in our simulations.

**Fig 3:**
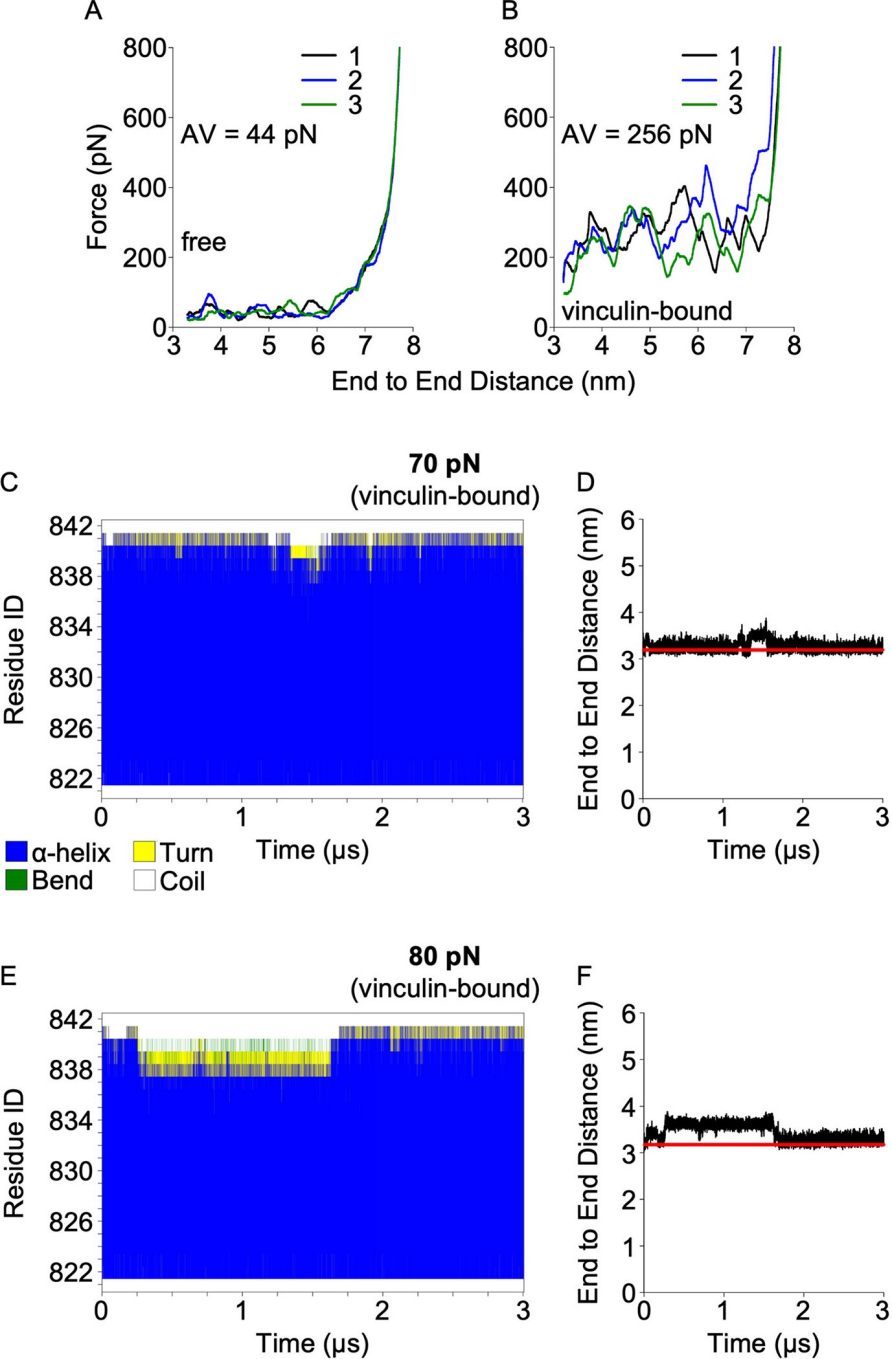
A vinculin-bound VBS can withstand high mechanical loading. (A,B) Unfolding force profiles for H11 in constant velocity SMD (A) H11 free and (B) H11 complexed with vinculin. Secondary structure evolution and end-to-end distance for H11 in constant force SMD at (C,D) 70 pN and (E,F) 80 pN force regimes. Red lines in panels D & F indicate the length of the vinculin-bound VBS at equilibrium conditions.

The unfolding force profiles demonstrate that vinculin binding strongly stabilizes the VBS. The free H11 and H12 both unfolded readily with an average unfolding force of 44 pN. In contrast, in the vinculin-bound state these VBSs unfolded at 256 pN and 243 pN respectively (Figs 3A, 3B, S4A, and S4B). While an isolated VBS experiences force-mediated conformational destabilization throughout the sequence (S5A-S5F Fig), vinculin binding protected the VBS core from force effects, resulting in more pronounced extension of the terminal residues only (S5G-S5L Fig). Overall, these findings confirm that the previously observed mechanical stabilization of talin rod fragments by vinculin is indeed mediated by stabilization of individual VBSs [22,25–27].

Constant velocity SMD is known to overestimate the unfolding force magnitude [19,20], and the measured unfolding forces were approximately one order of magnitude higher when compared to the force required experimentally for vinculin dissociation from talin (above 25 pN) [22,26]. Therefore, to reach unfolding force magnitude within the force range seen in previous studies (<100 pN), we performed microsecond-scale constant force SMD simulations. Using constant force at 70 pN, we achieved limited but noticeable VBS destabilization in the vinculin-bound state within 3 μs. Increasing the force to 80 pN led to a slight increase in terminal uncoiling of the VBS and a modest extension of the end-to-end distance (Figs 3C–3E and S4C–S4E), but no vinculin dissociation events were observed in this microsecond timeframe.

### Partially unfolded VBS refolds against mechanical load upon vinculin binding

According to recent reports, vinculin binding to talin triggers VBS coil-to-helix transition, observed as a reduction of the end-to-end distance of talin under force using magnetic tweezers [26]. However, the structural mechanism underlying vinculin–VBS binding when talin is under mechanical load remains to be elucidated. Thus, we next wanted to test whether vinculin a) facilitates VBS refolding against tension, or b) binding occurs only to the refolded VBS conformation. To study this, we performed equilibration of vinculin-bound VBS under 20 pN force, starting from a partially unfolded conformation (extended by 2 nm with ∼45% helicity).

**Fig 4:**
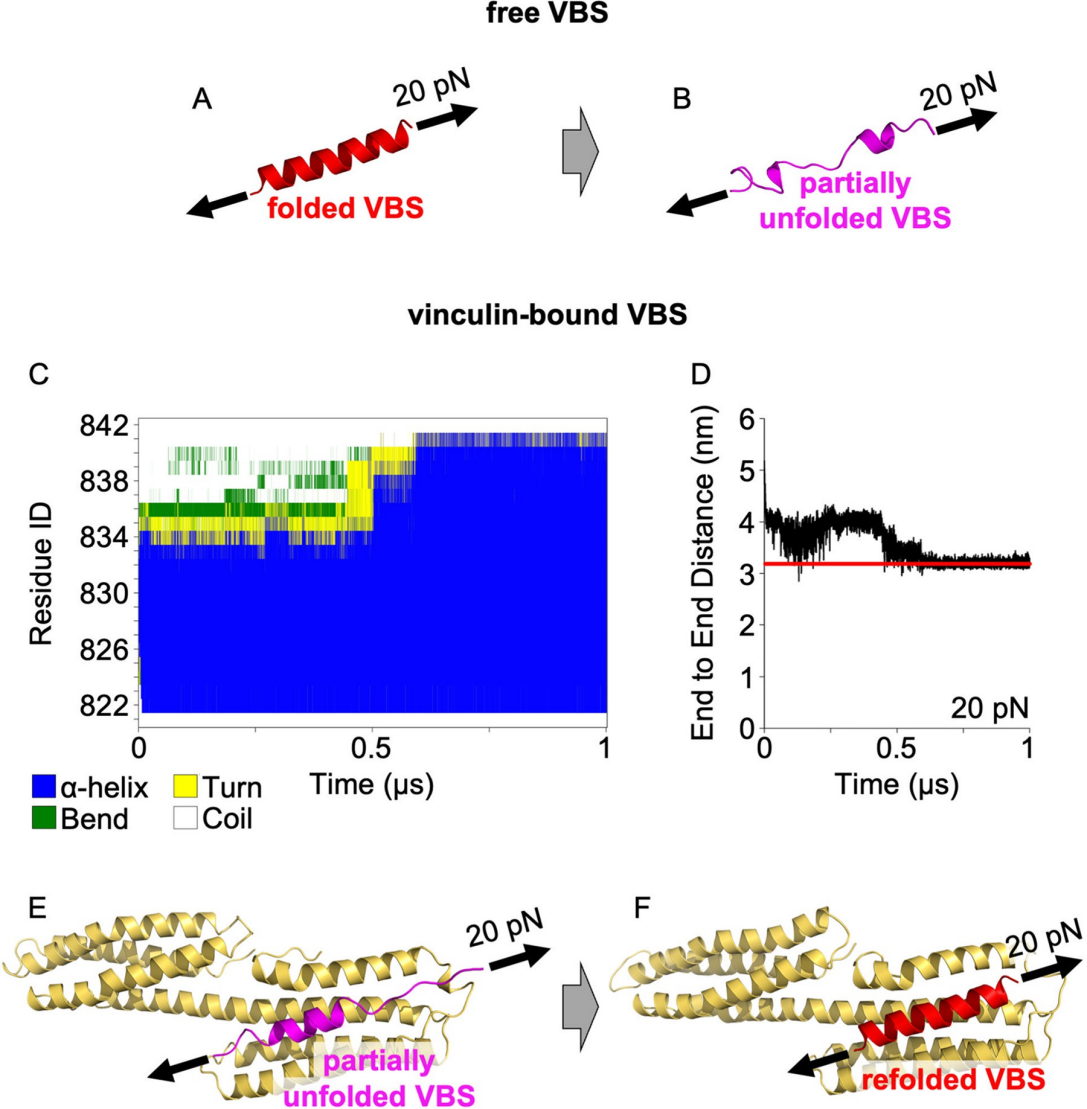
Vinculin binding promotes VBS refolding against mechanical load. Snapshots of (A) folded and (B) unfolded structural states of VBS captured at beginning and at 1 μs from SMD simulation using constant force at 20 pN. (C) Secondary structure evolution, (D) end-to-end distance and snapshots showing (E) starting and (F) final structures from SMD simulation using constant force at 20 pN, where a partially unfolded H11 R3 VBS complexed with vinculin refolds under mechanical loading. Secondary structure evolution and end-to-end distance plots for the free VBS SMD are shown in S2B Fig, replica 1. Red line in panel D indicates the average length of the refolded vinculin-bound VBS at equilibrium conditions.

As shown earlier, fast dynamics of a free VBS involves continuous unfolding and partial refolding under forces below 20 pN (Fig 1). In contrast, in the presence of vinculin, the VBS refolds efficiently into an α-helical conformation under 20 pN force within a timeframe of 0.3–1.3 μs (Fig 4), and vinculin protects the VBS from unfolding.

Both H11 and H12 showed almost identical behavior (S6 Fig). This result demonstrates that vinculin–VBS binding strongly stabilizes the α-helical VBS conformation and promotes refolding of the VBS helix by stabilizing the secondary structure against fluctuations induced by force.

### Vinculin binds partially unfolded VBSs

**Fig 5:**
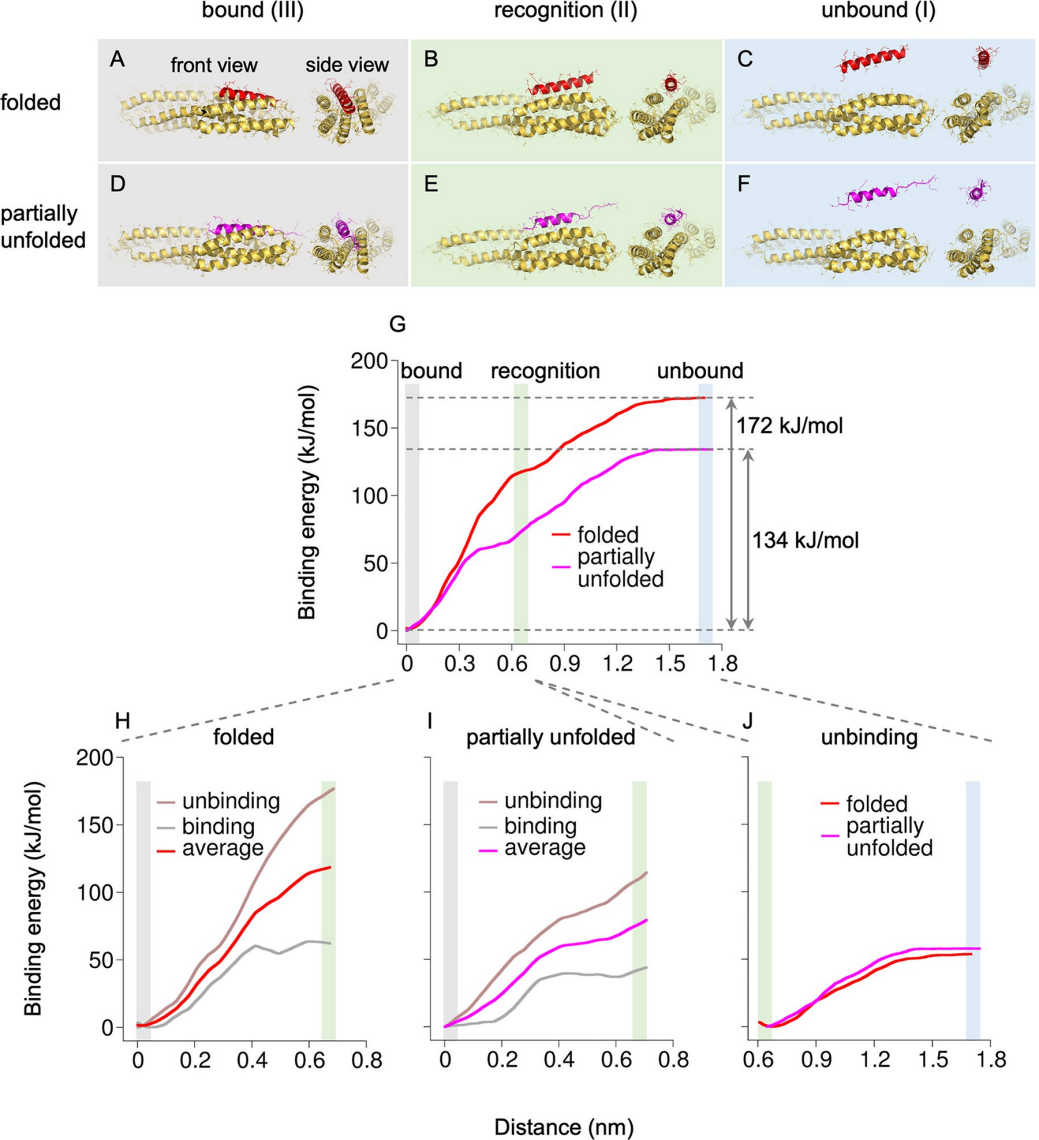
Vinculin binds partially unfolded VBS. (A-F) Secondary structure of folded (top, red) and partially unfolded (bottom, magenta) VBS in different states of binding; (A,D) complexed with vinculin via helix addition mechanism, (B,E) recognized by vinculin at the initial step of VBS-vinculin binding, and (C,F) VBS and vinculin are separated. (G) VBS-vinculin binding energy profile for folded and partially unfolded VBS, constructed as the average of the unbinding and binding profiles (H-J).

To assess the thermodynamic parameters of recognition and binding of partially unfolded talin VBS by vinculin, we used umbrella sampling and the weighted histogram analysis method [28] to calculate the vinculin–VBS binding energy profiles. We used two different starting states of VBS: 1) fully α-helical VBS and 2) partially unfolded VBS, stretched by 1.5 nm. This distance was chosen to be consistent with the previously observed contractions upon vinculin binding to R3 (∼1.5–2.5 nm per VBS, depending on force magnitude) [26]. The whole process of vinculin–VBS binding was divided into three main states: (I) unbound state, where the vinculin and the talin VBS are separated and do not interact; (II) recognition state – an intermediate state, where vinculin and VBS surfaces are in contact with each other but without major conformational changes in either protein at the initial step of binding; (III) bound state, where the VBS is completely embedded into vinculin via the helix-addition mode of binding (Fig 5A–5F).

Protein-protein binding energy is commonly calculated for the dissociation direction only, as it is straightforward to separate two molecules in simulations. However, using such an approach can overestimate the calculated binding energy as it does not account for the forward direction of the binding reaction. For this reason, to get higher confidence binding energy profiles for the “bound-recognition” part of the reaction (Fig 5G), we performed the calculation for both binding and unbinding directions (Fig 5H and 5I). This part of the binding process is crucial for the vinculin–VBS complexation, as complexation involves major structural rearrangements of vinculin helices due to the helix-addition mode of binding. The other part, “recognition-unbound”, was analyzed for the unbinding direction only (Fig 5J). The binding direction was not feasible due to complexity of initial protein-protein recognition that requires adjustment of sidechain orientation of interacting residues at the protein surface, including the Q19 sidechain (see S1 Text and S7 and S8 Figs), which is needed for the proper recognition. Although this limitation slightly hinders the calculated final binding free energy profiles, we assume that unbinding free energy for the “recognition-unbound” part calculated for the reverse direction is similar to the binding energy for the opposite, forward, direction, because this part of the reaction does not involve major structural rearrangements in either protein.

Strikingly, the final vinculin–VBS binding energy profiles suggest that vinculin recognizes a partially unfolded talin VBS similarly to a fully folded VBS helix at the initial step of binding (Fig 5G). However, the total binding energy for a fully folded VBS is higher (172 kJ/mol) than that measured for the partially unfolded VBS (134 kJ/mol). This is due to the significant difference in the binding energy at the “recognition-bound” stage, where a fully folded VBS helix embeds into vinculin much more strongly than a partially unfolded VBS.

We note that in our umbrella sampling simulations, the VBS conformation was restrained to keep the structure constant and to compare partially unfolded to fully folded VBS during the whole binding process. See details in Materials and Methods section.

## Discussion

Previous studies have shown that mechanical loading applied to talin enhances vinculin binding [17,22,25,26]. However, structural insights into the vinculin–VBS recognition remain elusive. Open questions include: how much VBS helix unfolding is allowed before it can no longer be recognized by vinculin, and what are the initial stages of how a mechanically exposed VBS becomes recognized by vinculin? These questions are especially pertinent if talin rod domain unfolding is not immediately followed by vinculin engagement, as the exposed VBS will be subject to a changing mechanical landscape. These questions are challenging to answer, in part due to the limitations in currently available structural methods, which cannot resolve protein structures when they are under tension. Although force stabilizes unfolded or partially unfolded protein conformations, a recent study shows that such conformational states are highly dynamic [29]. Unlike folded states of globular proteins, these unfolded conformations are high energy states stabilized by mechanical loads. They likely do not have specific conformations, and they continuously try to overcome the tension and fall into the low energy folded conformations. In addition, mechanical forces in the cell are not homogeneous [30], which further enhances fluctuations of the mechanically unfolded proteins. Partially unfolded conformations could also reach less dynamic conformations known as intermediate states [19,31].

Studies on protein unfolding by mechanical load, including talin rod domains, generally refer to “unfolded protein conformation” as the absence of tertiary structure. However, the conformations at the level of local secondary structure remain poorly understood, in part because the timescales of these processes are too fast. Computational methods are in a key position to visualize these early events and allow understanding of these processes in greater detail. As we present here, SMD allows the dynamics of individual VBS helices on the nanosecond to microsecond timescale to be visualized, revealing processes that are faster than the time resolution of experimental single molecule approaches. Our results highlight how SMD can be used to both recapitulate the experimental results computationally, which validates the approaches used, but then also go one step further and visualise the states and events that occur beyond the temporal resolution of experimental approaches.

Talin rod domains are solely formed of α-helices [11,32], and our results suggest that physiological forces allow dynamic reformation of α-helices. Depending on the force magnitude, some amount of secondary structure (α-helicity) frequently reforms in isolated talin VBS peptides, resulting in dynamic partial unfolding/refolding of α-helices.

Our study focuses on behaviour of individual VBS helices H11 and H12 when they are released from interactions with the other helices of talin rod domain R3 upon unfolding under force. In the context of full talin rod domains, the VBS dynamics and vinculin binding may be affected by possible interactions with other helices of talin and possible refolding, especially at low force. Talin R3 unfolds under approx. 5 pN [22], while individual VBS helices dynamically refold at significantly higher forces, allowing vinculin recruitment.

Our simulations visualize transitions between folded and unfolded conformations of a VBS helix under 10–20 pN within a few microseconds. In contrast, the temporal resolution of magnetic tweezers instruments is approx. 0.6–1 milliseconds [26,31], which is likely too low to detect the fast VBS dynamics that we observe in MD, probably revealing only discrete states with lifetime of milliseconds to seconds.

The “IVVI” mutant in R3 (R3^IVVI^) that was designed to stabilize the R3 domain [11] hops continually between its folded and unfolded state at 9 pN [26,31]. This constant unfolding/refolding behavior, observed as end-to-end distance fluctuations within ∼20 nm, demonstrates that not only α-helix refolding, but also huge (∼20 nm) distance contractions to pack the helices together to form the R3 domain tertiary structure can occur under tension. In contrast to a whole domain, refolding of individual α-helices does not require major distance contraction. Our simulations suggest that they dynamically fluctuate between folded and unfolded states at even higher forces than 9 pN, forming partially unfolded/refolded α-helical conformations, which vinculin can recognize and bind. Local refolding of VBS peptides enables vinculin to recognize them, preventing irreversible mechanical unfolding of talin.

Higher forces (above 25 pN) on talin markedly accelerate vinculin dissociation from talin [22,26], observed as an end-to-end distance increase of ∼3 nm per VBS as the helix unravels to a random coil. Similarly, vinculin–talin binding was recently found to cause talin’s end-to-end distance contraction upon vinculin binding, and the length of contraction increases with force [26]. Likewise in our SMD simulations, VBS also forms more extended conformations under higher forces.

Vinculin–VBS binding occurs within hundreds of milliseconds under lower forces, while above 15 pN, it is a fast transition [26]. In our SMD simulations, we observed significant VBS bending and formation of compact conformations under low force (10 pN), which may result in VBS autoinhibition [27] or other local misfolding, especially in the context of the larger protein, that may not occur in the isolated VBS. Low force allows refolding of α-helices, but does not efficiently align the unstable VBS peptide to prevent it from forming autoinhibited/misfolded compact conformations, which may explain the previously observed slow vinculin binding at lower forces [26]. In contrast, higher physiological forces (above 15 pN) prevent the VBS from forming autoinhibited compact conformations, while still allowing dynamic, but significant VBS refolding, which enables vinculin to recognize and refold the VBS peptide. In other words, forced constraining of VBS primes it for vinculin binding.

Taken together, our data supports a model where exposed VBSs are predominantly unstructured under force but exhibit rapid spontaneous local refolding. Vinculin can recognize such partially refolded VBS helices. Upon recognition, vinculin strongly stabilizes the VBS and facilitates VBS refolding, embedding the VBS as a fifth helix within vinculin VD1, to form a high affinity complex (see S1 Video). This recognition property allows vinculin–talin complexation at different force regimes, enabling formation of mechanical load reinforcing focal adhesions, where the vinculin–talin complex is one of the key elements that are necessary for their growth and strengthening [33,34]. The proposed model of protein–protein recognition and binding under force may be a general mechanism for force-modulated interactions, where fluctuations in forces allow dynamic formations of partially unfolded structures required for initial recognition.

## Materials and methods

### Structure preparation and analysis

In all simulations, the talin protein segment spanning residues 821–842 for the R3 VBS in helix 11 (H11) and residues 854–875 for the R3 VBS in helix 12 (H12) were used. Vinculin head domain 1 (VD1), residues 1–252, was used in all simulations. Starting structures used in MD for the R3 VBSs were adopted from the NMR structure of talin R3, 2L7A [11]. Vinculin complexed with H11 and H12 were adopted from the crystal structures 1ZVZ and 1U6H respectively [12,35]. Free vinculin was prepared using the 1RKE crystal structure [14]. Acetyl (ACE) and N-methyl (NME) capping groups were used for both termini of the VBS peptides and C-terminus of vinculin construct to avoid charged residues. Analysis of the protein secondary structure evolution was performed using DSSP [36].

First microsecond of 50-microsecond long SMD simulations was considered as initial unfolding of starting folded VBS conformation and was omitted from analysis.

### MD simulations

All MD simulations were performed with Gromacs 2021 [37] using Amber14SB force field [38], which was proved to be one of the best for studying of protein peptides [39]. SPC/E water model in 0.15 M KCl [40] was used. The systems were energy minimized and then equilibrated using harmonic position restraints on all heavy atoms of the protein. The Berendsen algorithm [41] was used to control both temperature and pressure during the system equilibration phase. The temperature and pressure of the system was maintained at 300 K and 1 bar using V-rescale [42] and Parrinello-Rahman [43] algorithms. The temperature coupling was applied separately for the protein and the solution parts. Integration time step of 2 fs was used in all simulations. Three independent MD trajectories were generated for each system.

Computational resources were provided by CSC–IT Center for Science, Finland.

### SMD simulations

Steered molecular dynamics on VBS unfolding was performed by extending the VBS’s end-to-end distance using the C-alpha atoms of the terminal residues for pulling. Constant velocity pulling at 0.01 nm/ns and spring constant at 1000 kJ/mol nm^2^ were used for both free and vinculin-bound VBSs. In constant velocity pulling simulations the pressure was maintained using the Berendsen algorithm [41] and was switched off for the direction of pulling. Average unfolding force was calculated for end-to-end distance extension range up to ∼6.4 nm.

Constant force pulling at 70 pN and 80 pN in 3 μs simulations was used for vinculin-bound VBS. 1 μs simulations with constant force at 15 pN and 20 pN were performed for free H11 R3 VBS, while 10 pN and 15 pN for free H12 R3 VBS. In this setup, VBS termini were unrestrained and fully flexible. Three independent MD trajectories were generated for 1 μs SMD simulations, while one for 3 μs SMD simulations.

Longer constant force simulations were performed with help of Plumed plugin, version 2.9.0 [44]. In this setup, 10 pN, 15 pN and 20 pN force was used with H11 R3 VBS. To reduce excessive flexibility of VBS termini and mimic conditions where the VBS peptide is located within the larger protein, movements of VBS termini were restrained using soft harmonic potential restraints at 40 kJ/mol nm^2^, applied as restraint of XY distance components between terminal C-alpha atoms.

### Binding free energy calculations using umbrella sampling

The free energy for vinculin–VBS binding was calculated using umbrella sampling and the weighted histogram analysis method [28]. It involves defining the binding/unbinding process (reaction coordinate) as the distance between two molecules. The reaction coordinate is then divided into multiple “intermediate conformations” (umbrella windows) covering the entire process with a certain distance step. This distance between neighboring windows is referred to as the “window size.” It is crucial for the window size to be small enough to accurately build a free energy profile later. During MD equilibration, these umbrella windows are restrained at the selected distance using a harmonic force constant. The force constant should be stiff enough to keep the distance close to the initially selected value for the window, yet not too stiff because it must allow overlapping with neighboring windows. This approach ensures that the entire reaction coordinate is adequately sampled. Finally, the data is processed using the weighted histogram analysis method to construct the free energy profile. In summary, the choice of window size and harmonic force constant depends on the strength and nature of the interactions, with smaller window sizes generally yielding better results.

The whole vinculin–VBS binding reaction pathway was defined by three states; “bound”, “recognition”, and “unbound”. Hence, the binding energy was calculated in two parts, “bound–recognition” and “recognition–unbound”. For the “bound–recognition” part, the umbrella sampling calculations were performed for both directions of the reaction, unbinding and binding, and the average value was obtained.

The reaction coordinate was defined as a center-of-mass distance between backbone atoms of the VBS and backbone atoms of vinculin helices in the vicinity of the talin binding site, spanning residues 6–25 (H1), 39–60 (H2), 73–94 (H3), 104–126 (H4). The VBS conformation and rotations were restrained in all dimensions using soft position restraints with harmonic potential at 40 kJ/mol nm^2^ for backbone heavy atoms to keep its conformation constant and avoid unwanted rotations during the binding/unbinding process. The end-to-end distance of partially unfolded VBS was restrained at 4.7 nm using harmonic potential restraint at 2000 kJ/mol nm^2^. Rotations of vinculin were restrained using soft position restraints with harmonic potential at 40 kJ/mol nm^2^ for backbone heavy atoms of H3 (residues 67–96) and H4 (residues 101–129) in the xy-plane only, allowing its movement in z-dimension to bind/unbind the VBS. Importantly, positions/movements of H1 and H2 in vinculin were not restrained to allow them to adopt proper conformations upon the VBS binding/unbinding. The starting structures for umbrella sampling were taken from the corresponding SMD trajectories on VBS binding/unbinding using constant velocity pulling at 0.05 nm/ns and spring constant at 2000 kJ/mol nm^2^. The crystal structure of vinculin complexed with H11 R3 VBS (1ZVZ) [12] was used as a starting conformation for the unbinding reaction, while free vinculin with the H11 R3 VBS manually positioned on the vinculin’s surface (recognition state) was used for the SMD of vinculin–VBS binding. Umbrella sampling was performed using 17 windows for each system with 0.04 nm window size, 100 ns duration and the umbrella was set to 18000 kJ/mol nm^2^. The umbrella sampling calculations for the “bound–recognition” part were repeated four times and the average free energy was obtained.

The “recognition–unbound” part of the binding reaction pathway was analyzed in the unbinding direction only, where 22 windows were linearly spaced between these two states. Window size was 0.05 nm, duration was 100 ns, and the umbrella was set to 15,000 kJ/mol nm^2^. For this part, the umbrella sampling calculations were repeated six times and average free energy was obtained.

Umbrella windows size and harmonic force constant were always selected based on preliminary testing, making sure to have sufficient overlap between the distributions of the restrained distance in neighboring replicas.

In this analysis, we used H11 R3 VBS only, because the analysis is demanding and computationally expensive.

### Protein expression and purification

The VD1 domain of mouse vinculin (mVD1) was obtained as a codon-optimized synthetic gene in pET151 (GeneArt). The Q19S point mutation was introduced by site-directed mutagenesis and the sequence was verified.

For expression of VD1 and VD1(Q19S), BL21(DE3) competent cells were transformed with the relevant plasmid and grown in lysogeny broth, supplemented with 100 μg/mL ampicillin at 37°C until the OD at 600 nm reached ~0.6. Protein expression was induced by the addition of 0.4 mM IPTG and expression proceeded overnight at 20°C. Cells were harvested by centrifugation, resuspended in lysis buffer (50 mM Tris-HCl pH 8, 250 mM NaCl, 5% v/v glycerol) at 5 mL per gram of cells and stored at -80°C.

Proteins were purified by nickel-affinity chromatography, followed by anion exchange chromatography. Briefly, cells were thawed, supplemented with 1 mM PMSF and 0.2% v/v Triton X-100 and lysed by sonication. Cell debris were removed by centrifugation and the supernatant was filtered and loaded onto a 5 mL HisTrap HP column (Cytiva) using an AKTA Start (GE Healthcare). The column was washed with buffer containing 50 mM Tris-HCl pH 8, 600 mM NaCl, 30 mM imidazole, 4 mM MgCl_2_, 4 mM ATP, 5% v/v glycerol, 0.2% v/v Triton X-100 and bound protein was eluted across a 75 mL linear gradient of 0–300 mM Imidazole. Fractions containing mVD1 were pooled, supplemented with AcTEV protease (Invitrogen) to remove the His-Tag and dialyzed against 20 mM Tris-HCl pH 8, 50 mM NaCl. Protein was loaded onto a 5 mL HiTrap Q (Cytiva), eluted across a 75 mL linear gradient of 50–750 mM NaCl, dialyzed against phosphate buffered saline (PBS) pH 7.4, snap-frozen in liquid nitrogen and stored at -80°C.

### Circular dichroism (CD)

Circular dichroism was performed using a JASCO J-715 spectropolarimeter with 0.45 mg/mL protein samples in PBS pH 7.4. The far-UV spectra were an average of 6 scans collected between 195 and 300 nm, at 50 nm/min, with a 1 nm step resolution and a bandwidth of 1 nm. CD melting curve data was collected between 20 and 90°C at a wavelength of 222 nm, with a 1°C step resolution and 1 nm bandwidth.

### Size exclusion chromatography (SEC)

Samples of 100 μL protein at 100 μM protein concentration were injected onto a Superdex G-200 increase 10/300 (GE Healthcare) at 0.75 mL/min in PBS pH 7.4 (supplemented with 5 mM DTT) using an AKTA Pure (GE Healthcare), monitoring absorbance at 280 nm.

### Fluorescence polarisation (FP)

The mouse talin1 R1 VBS (H4) peptide (C-RPLLQAAKGLAGAVSELLRSA) was synthesized by GLBiochem (Shanghai) with a non-native, N-terminal cysteine residue. The peptide was coupled with a maleimide-fluorescein dye (Thermo Fisher Scientific) following the manufacturers protocol. Assays were performed in triplicate with a 2-fold serial dilution of protein, with VBS peptide at 700 nM. Fluorescence polarization was measured using a CLARIOstar plate reader (BMGLabTech) at 25°C (excitation: 482 ± 8 nm; emission: 530 ± 20 nm). Data were analyzed using GraphPad Prism 8 software and K_d_ values were generated using the one-site total binding equation.

### Cell biology

Vinculin-null mouse embryonic fibroblasts (MEF) were a kind gift from Prof. Carsten Grashoff (University of Münster) and have been previously described [3]. Cells were maintained at 10–80% confluency in high-glucose Dulbecco’s modified Eagle medium with GlutaMax (10566016, Thermo Fisher Scientific) supplemented with 10% fetal bovine serum (Gibco) in a humidified 37°C, 5% CO_2_ incubator. For FRAP experiments, Vin-/- MEF cells were adapted to FluoroBrite DMEM medium (Thermo Fisher Scientific) supplemented with 10% fetal bovine serum and 1% GlutaMax for a minimum of 48 hours.

### Expression constructs and cell transfection

mEmerald-Vinculin-N-21 was a gift from Michael Davidson (Addgene plasmid # 54304; http://n2t.net/addgene:54304; RRID:Addgene_54304). To restore the wildtype human vinculin sequence, L234V backmutation was introduced to all vinculin constructs. Q19S mutation was introduced by GenScript cloning service. All plasmid constructs were authenticated by sequencing. Vin-/- MEF cells were transfected with the Neon transfection system (Thermo Fisher Scientific). For all plasmid constructs, 5 μg plasmid DNA was electroporated to 10^6^ cells using 1300 V, 30 ms and 1 pulse.

### Immunostaining and confocal imaging

Zeiss high-performance 170-μm-thick coverslips were washed with 2% Hellmanex-III (Merck) in a bath sonicator at 40°C for 20 min (Finnsonic), rinsed with deionized water and attached to perforated 35 mm polystyrene dishes (MatTek). The coverslips were coated with 25 μg/ml human fibronectin in PBS pH 7.4 for 30 min at 37°C and washed twice with PBS. Vin-/- MEF cells were transfected with vinculin expression constructs as described above and allowed to recover for 24 hours. Transfected cells were trypsinized and plated on fibronectin-coated coverslips for 8 hours, after which medium was aspirated and the cells fixed with 4% paraformaldehyde (PFA) in PBS for 20 min at 22°C (RT). The cells were washed 3 times with PBS and permeabilized with 0.2% Triton X-100 in PBS for 5 min at RT. Non-specific antibody binding was blocked with 5% fetal calf serum, 1% bovine serum albumin (BSA) and 0.05% Triton X-100 in PBS for 30 min at RT. Polyclonal rabbit anti-talin 1 (Abcam ab71333, RRID: AB_2204002) was diluted 1:450 in the blocking buffer and incubated for 60 min at RT. Cells were washed 4 times with PBS. Goat anti-rabbit AlexaFluor-568 was used as secondary antibody (Thermo Fisher Scientific A11011, RRID: AB_143157) at a dilution of 1:250 together with Phalloidin-iFluor647 (Abcam ab176759) at a dilution of 1:330 in the blocking buffer and incubated for 90 min at RT. Cells were washed 4 times with PBS and stored at 4°C.

For adhesion intensity analysis, immunostained cells were imaged using a Nikon Eclipse Ti2-E inverted microscope equipped with A1R+ laser scanning confocal unit (Nikon) and Nikon CFI Plan Apo IR SR 60x WI (NA 1.27) objective. For both studied vinculin variants, 22–23 images with 140 nm pixel size and 200 nm Z-stack step size were captured with identical imaging parameters. Talin and vinculin intensities in adhesion sites were analyzed using Fiji distribution of ImageJ 1.53t [45]. A binary mask created by vinculin channel thresholding was used to measure mean vinculin and talin intensity values and to calculate cell-specific vinculin/talin intensity ratios. For each image, the Z-stack slice with the highest adhesion-to-cytosol contrast was used in the analysis.

### Live-cell imaging and FRAP experiments

35 mm glass-bottom dishes were prepared and fibronectin coated as described above. Transfected Vin-/- MEF cells were allowed to recover for 24 hours, trypsinized (TrypLE Express, Thermo Fisher Scientific) and plated on fibronectin-coated glass-bottom dishes in complete FluoroBrite media (FluoroBrite DMEM + 10% FBS + 1% GlutaMax). Cells were allowed to attach for 60 minutes, media replaced and the dish mounted to a humified 37°C, 5% CO_2_ incubator on the microscope stage. A Nikon Eclipse Ti2-E inverted microscope equipped with A1R+ laser scanning confocal unit (Nikon), Perfect Focus System (Nikon) and Nikon CFI Plan Apo IR SR 60x WI (NA 1.27) objective was used for all FRAP experiments. Cells were imaged at 488 nm wavelength (0.7% laser) and 210 nm pixel size (53.76 × 53.76 μm field, 256x256 resolution) for 10 pre-bleach images at 1 second intervals. A circular region of 5.0 μm was photo-bleached using 80% 488 nm laser, scanning for 3 rounds at 8 fps (total 350 ms) and fluorescence recovery immediately followed for 150 seconds at 2 second intervals with settings as for the pre-bleach images. For each studied cell, only one adhesion was photobleached. For FRAP image analysis, 15 images from two independent experiments were analyzed using EasyFrap software [46] with double-normalization and single-term equation fit to determine vinculin t_half_-values and mobile fractions.

### Peptide biotinylation

Solid-phase synthesized talin1 H12 R3 (C-DQLLEAARNLSSAFSDLLKAA) with free N-terminal cysteine was labelled with maleimide-PEG2-biotin (Thermo Fisher Scientific A39261). Lyophilized peptide was dissolved in PBS pH 7.4 and reduced at 100 μM peptide concentration with 250 μM TCEP in PBS supplemented with 0.25% Triton-X100 for 10 min at RT. Maleimide-PEG2-biotin was added at 5-fold molar excess (500 μM) and incubation continued for 2 hours at RT, mixing at 30 rpm. The reaction was quenched with β-mercaptoethanol added at 5-fold molar excess over maleimide-PEG2-biotin (2.5 mM) for 20 min at RT. Biotin-labelled peptide was purified using a PD10 desalting column (G25 resin, Cytiva Life Sciences) equilibrated with PBS. Elution fractions containing biotinylated peptide were identified using PageBlue-staining (Thermo Fisher Scientific) of glutaraldehyde-fixed (5%, 2 hours) Bolt BisTris Plus 4–12% SDS-PAGE gels (Thermo Fisher Scientific). The concentration of pooled elution fractions was determined using Pierce BCA microplate assay (Thermo Fisher Scientific) measured with Victor Nivo at 562 nm (PerkinElmer). Aliquoted peptide was flash-frozen with liquid nitrogen and stored at -80°C.

### Biosensor experiments

The binding and dissociation kinetics of recombinant WT and Q19S vinculin VD1 to talin1 H12 R3 were determined using ForteBio Octet RED384. Streptavidin tips (18–5019, Sartorius) were rehydrated in kinetic buffer (50 mM Tris-HCl pH 7.4, 150 mM NaCl, 2 mM TCEP, 0.02% Tween, 0.1% BSA) for 90 minutes. To avoid mass transfer effect due to dense sensor functionalization with ligand, 2 μM biotinylated H12 R3 in kinetic buffer was mixed with 14 μM biocytin (Sigma Aldrich) before peptide loading on the sensor tips for 600 seconds, after which the remaining binding sites were quenched with 26 μM biocytin for 180 seconds. Wildtype and Q19S VD1 were diluted with kinetic buffer with a 6-point 4-fold dilution series ranging from 16 μM to 15.6 nM and allowed to associate with the immobilized peptide for 600 seconds. The dissociation of sensor-bound VD1 was followed for 1200 seconds. All incubations were performed at 28°C with shaking at 500 rpm. Control samples without biotinylated H12 R3 peptide or VD1 were used to normalize the results. Global fits across the VD1 concentration series were used to determine kinetic parameters for the binding of both vinculin forms.

## Supporting information

S1 Text Q19 in vinculin head sterically hinders VBS binding. (PDF)

S1 Video Vinculin binds partially unfolded VBS and facilitates its refolding under 20 pN force. Upon recognition, partially unfolded H11 R3 VBS becomes embedded as a fifth helix within vinculin VD1, and shows efficient refolding under 20 pN force applied to the termini of the VBS. Vinculin is shown in gray and VBS in magenta.
(AVI)

S1 Fig Force-free equilibration of isolated VBS. Secondary structure evolution, end-to-end distance, and intermediate snapshots for VBSs, (A,C) H11 R3 and (B,D) H12 R3. Three independent replicas were performed.
(TIF)

S2 Fig Equilibration of isolated VBS under mechanical loading. Secondary structure evolution, end-to-end distance, and final snapshot in SMD simulations using constant force at (A) 15 pN and (B) 20 pN for H11 R3 VBS, and (C) 10 pN and (D) 15 pN for H12 R3 VBS. Three independent replicas were generated for each setup.
(TIF)

S3 Fig Vinculin-bound VBS is stable in equilibrium MD. Secondary structure evolution, end-to-end distance, and final snapshot for both, (A) H11 R3 VBS and (B) H12 R3 VBS complexed with vinculin in equilibrium (force-free) MD simulations. Three independent replicas for each system were generated.
(TIF)

S4 Fig Vinculin-bound VBS withstands high mechanical loading. Unfolding force profiles for H12 R3 VBS in constant velocity SMD for (A) free and (B) complexed with vinculin. Secondary structure evolution and end-to-end distance for H12 R3 VBS in constant force SMD at (C,D) 70 pN and (E,F) 80 pN force regimes.
(TIF)

S5 Fig VBS unfolding in both free and vinculin-bound state. Secondary structure evolution plots for free and vinculin-bound (A-C, G-I) H11 R3 VBS, and (D-F, J-L) H12 R3 VBS. Three independent replicas were generated for each system.
(TIF)

S6 Fig Partially unfolded VBS refolds under 20 pN force upon vinculin binding. Secondary structure evolution and end-to-end distance for (A,B,E,F,I,J) H11 R3 VBS and (C,D,G,H,K,L) H12 R3 VBS in constant force SMD at 20 pN. Replica 2 (E,F) is shown in Fig 4C and 4D and is highlighted with dashed line box.
(TIF)

S7 Fig Q19 in vinculin head sterically hinders VBS binding. (A-B) Cartoon representation of VD1 tertiary structure, the Q19 side chain is shown in blue and the talin binding site in orange. (C-D) Superimposition of 1000 structure snapshots captured at every 1 ns from the 1 μs MD trajectory showing conformations of the Q19 sidechain. VD1 helices are shown as ribbons and Q19 sidechain as lines with the starting Q19 conformation in blue and the final confirmation in red. Superimposition performed using C-alpha atoms of Q19 and neighboring residues in the vinculin helices, H1 and H2. (E) Representative closed and open conformations of the Q19 sidechain shown as sticks.
(TIF)

S8 Fig Q19 might act as a gatekeeper for vinculin–VBS recognition and the initial step of binding. (A) Circular Dichroism (CD) thermal denaturation profiles of mVD1. Protein at 0.45 mg/mL was thermally denatured and the change in CD was monitored at 222 nm. The Tm of WT (blue) and Q19S (red) are shown in parentheses. (B) Oligomeric state analyzed by size exclusion chromatography. 100 μL of protein at 100 μM was loaded onto an S200 Increase G10/300 column and the absorbance was monitored at 280 nm. Both WT (blue) and Q19S (red) were monomeric in solution. Curves are offset by 1 mAU for clarity. (C) Fluorescence Polarization assay of WT (blue) and Q19S (red) protein binding a fluorescein labelled Tln1 H4 peptide. mVD1(Q19S) binds Tln1 H4 with a ~1.75-fold higher affinity than WT mVD1. The K_D_ is shown in parentheses. (D) Representative images of vinculin-mEmerald or Q19S mutant localization in vinculin knock-out fibroblast cells. For individual channels, inverted fluorescence signal is shown. Merge image channel coloring is indicated by the colored bars above each individual channel. (E) Adhesion vinculin/talin intensity ratio in images of vinculin-mEmerald and anti-talin antibody staining. Each dot represents mean intensity ratio for a single cell. n = 22 and 23 cells for vinculin-mEmerald and Q19S mutant, respectively. (F) FRAP analysis of vinculin dynamics in vinculin knock-out fibroblast cells. Mean ± 1 SD. n = 15 cells for both vinculin variants, pooled from two independent experiments. (G-H) Mobile fraction and recovery half-time for FRAP data in F using a single-term curve fit. Mobile fraction of 1.0 indicates complete recovery of fluorescence signal. (I) Biosensor analysis of VD1 binding to biotinylated and immobilized on streptavidin-functionalized sensor talin H12 helix. WT vinculin head and Q19S mutant were incubated with H12-functionalized sensor tips for 600 sec using a 4-fold concentration series, followed by a dissociation phase for 1200 sec. (J) K_D_, k_on_ and k_off_ values determined using global fit to biosensor data shown in I.
(TIF)
