# Supplementary material for: Molecular dynamics simulations reveal how vinculin refolds partially unfolded talin rod helices to stabilize them against mechanical force
Source: PLoS Comput Biol. 2024 Aug 7;20(8):e1012341. doi: 10.1371/journal.pcbi.1012341 (PMC11333002; doi:10.1371/journal.pcbi.1012341)
Supplement: S1 Text — (PDF) [file pcbi.1012341.s001.pdf]

Supporting Information

Molecular dynamics simulations reveal how vinculin refolds partially unfolded talin rod helices to stabilize them against mechanical force

Vasyl V. Mykuliak[1,*], Rolle Rahikainen[1¶], Neil J. Ball[2,3¶], Giovanni Bussi[4], Benjamin T. Goult[2,3], Vesa P. Hytönen[1,5,*]

[1]Faculty of Medicine and Health Technology, Tampere University, Tampere, Finland

[2]School of Biosciences, University of Kent, Canterbury, UK

[3]Present address: Department of Biochemistry, Cell & Systems Biology, Institute of Systems, Molecular & Integrative Biology, University of Liverpool, Liverpool, UK

[4]Scuola Internazionale Superiore di Studi Avanzati, SISSA, Trieste, Italy

[5]Fimlab Laboratories, Tampere, Finland

[¶] contributed equally

* vasyl.mykuliak@tuni.fi; * vesa.hytonen@tuni.fi

Q19 in vinculin head sterically hinders VBS binding

During the MD analysis of vinculin–VBS interactions we noticed that Q19 in VD1 had an impact on VBS binding. Q19 is located on the surface of VD1 near the talin binding site (S7A and S7B Fig) and certain orientations of the side chain sterically hinder VBS recognition by vinculin. Our simulations demonstrate that the Q19 sidechain is flexible, allowing multiple dynamic conformations (S7C and S7D Fig). Some of these conformations obstruct the VBS binding groove, indicating a swing out movement of the sidechain is required (S7E Fig) to enable complexation with the VBS via the helix-addition mechanism.

To gauge the extent of the potential steric hinderance of the large glutamine sidechain on the talin-vinculin complexation we designed a Q19S VD1 mutant. VD1(Q19S) expressed equally to the wild-type protein and showed high similarity in thermal stability with almost identical melting temperature, T_m (Q19S: 59°C; WT: 60°C) (S8A Fig). We analyzed the VD1–VBS binding affinity using a fluorescence polarization assay, that was previously used to study the VD1–VBS binding [1]. We observed that Q19S had a modest effect on the binding affinity (K_d = 203 nM), binding approximately 1.7-fold tighter compared to wild-type VD1 (K_d = 350 nM) (S8C Fig).

To evaluate whether the Q19S mutation might influence vinculin function in cells, we used vinculin-null fibroblast cells [2] transfected with vinculin constructs, and observed that full-length Q19S vinculin was incorporated into talin-rich focal adhesions similarly to WT vinculin (S8D and S8E Fig). We also measured the stability of the vinculin in focal adhesions using FRAP technology and observed similar exchange dynamics

(S8F-H Fig). Biosensor analysis with biolayer interferometry using immobilized VBS did not reveal significant differences in the binding or dissociation kinetics between WT and Q19S VD1 (S8I and S8J Fig).

Overall, our simulations indicate that residue Q19 might act as molecular gatekeeper in vinculin to control the entry of the VBS. While replacement of the residue had modest but measurable enhancement in binding affinity measured by fluorescence polarization, we did not observe a significant difference between cells expressing Q19S or wild-type vinculin, although subtle phenotypes can be hard to detect in a cultured system on glass. The impact of Q19 as a gatekeeper may be dependent on VBS conformation at the recognition step of vinculin–talin binding, where the position of the sidechain may have reduced impact for partially unfolded VBS conformations.

References

1. Wang Y, Yao M, Baker KB, Gough RE, Le S, Goult BT, et al. Force-Dependent Interactions between Talin and Full-Length Vinculin. J Am Chem Soc. 2021;143: 14726–14737. doi:10.1021/jacs.1c06223
2. Austen K, Ringer P, Mehlich A, Chrostek-Grashoff A, Kluger C, Klingner C, et al. Extracellular rigidity sensing by talin isoform-specific mechanical linkages. Nat Cell Biol. 2015;17: 1597–1606. doi:10.1038/ncb3268
