# Supplementary figures and images for: Molecular dynamics simulations reveal how vinculin refolds partially unfolded talin rod helices to stabilize them against mechanical force
Source: PLoS Comput Biol. 2024 Aug 7;20(8):e1012341. doi: 10.1371/journal.pcbi.1012341 (PMC11333002; doi:10.1371/journal.pcbi.1012341)

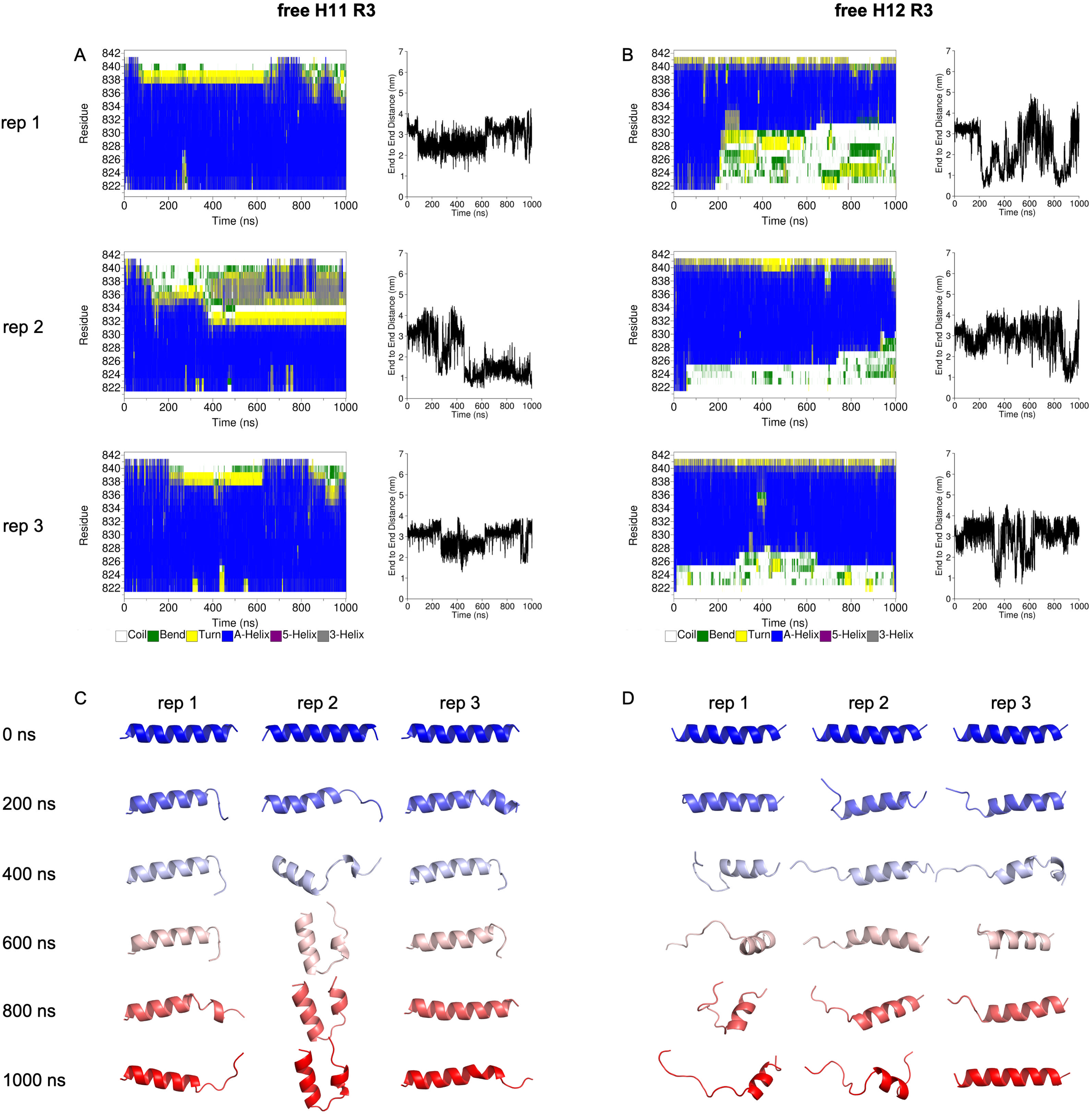

Supplement: S1 Fig — Secondary structure evolution, end-to-end distance, and intermediate snapshots for VBSs, (A,C) H11 R3 and (B,D) H12 R3. Three independent replicas were performed. (TIF) [file pcbi.1012341.s003.tif]

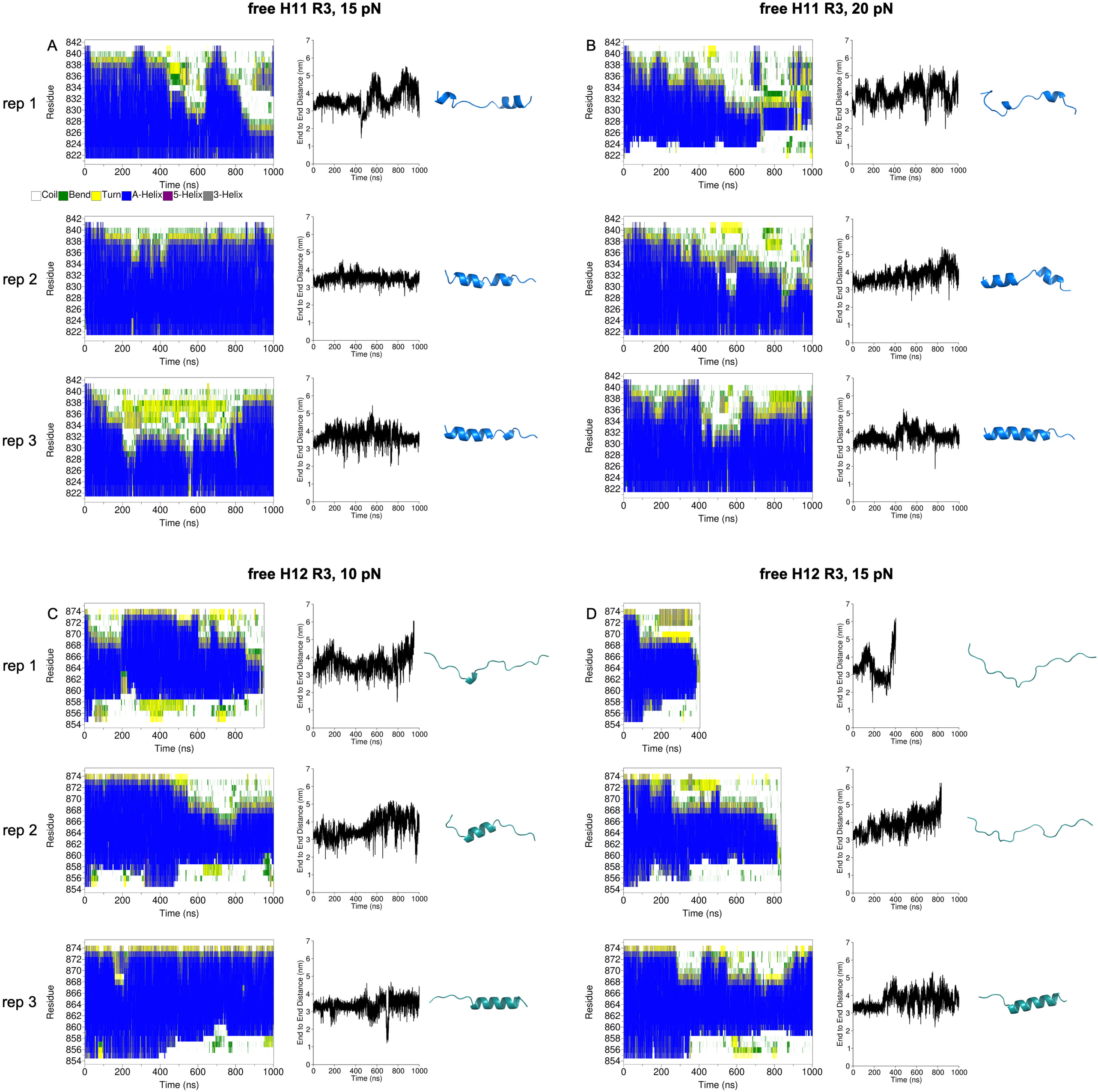

Supplement: S2 Fig — Secondary structure evolution, end-to-end distance, and final snapshot in SMD simulations using constant force at (A) 15 pN and (B) 20 pN for H11 R3 VBS, and (C) 10 pN and (D) 15 pN for H12 R3 VBS. Three independent replicas were generated for each setup. (TIF) [file pcbi.1012341.s004.tif]

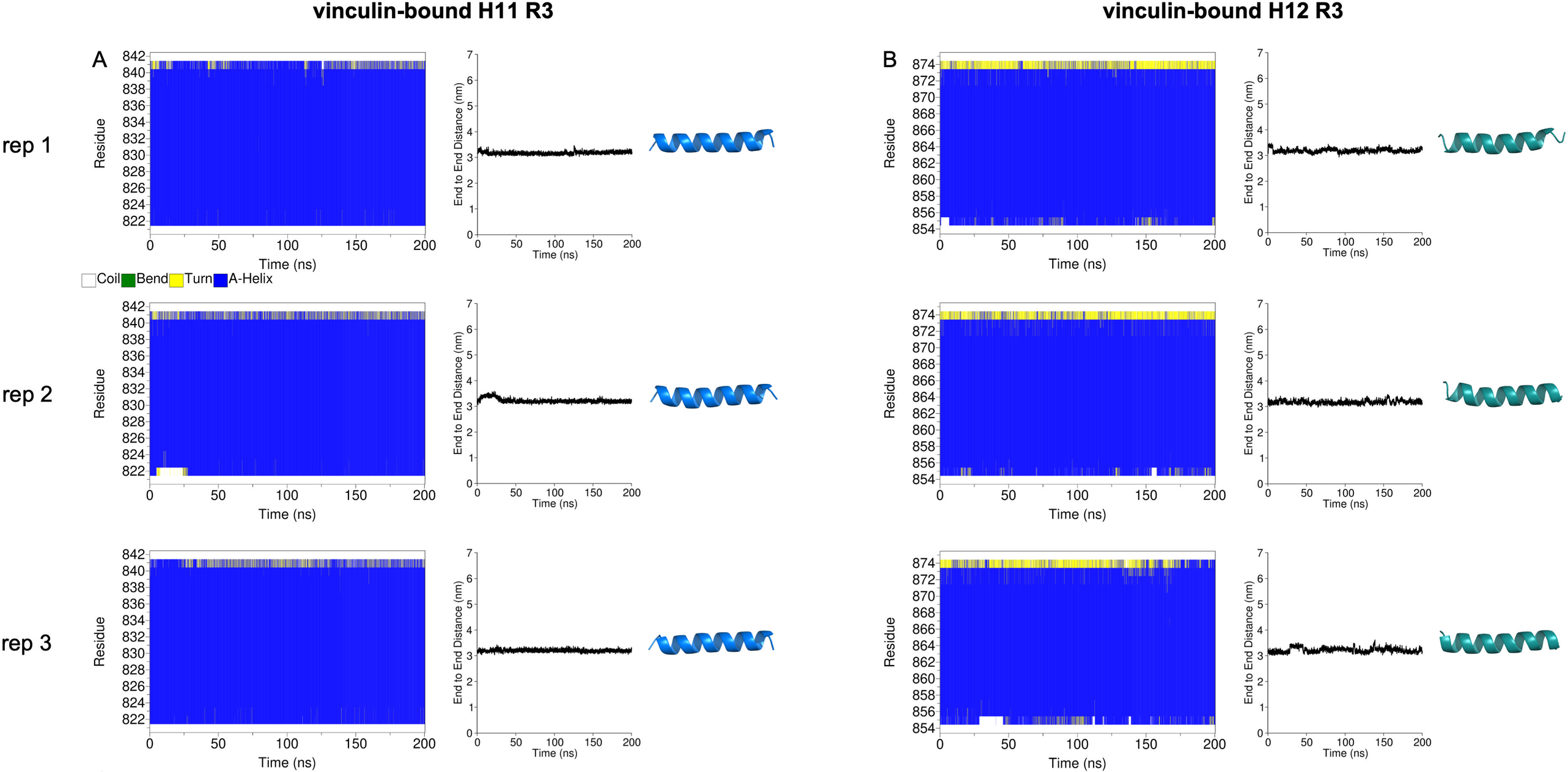

Supplement: S3 Fig — Secondary structure evolution, end-to-end distance, and final snapshot for both, (A) H11 R3 VBS and (B) H12 R3 VBS complexed with vinculin in equilibrium (force-free) MD simulations. Three independent replicas for each system were generated. (TIF) [file pcbi.1012341.s005.tif]

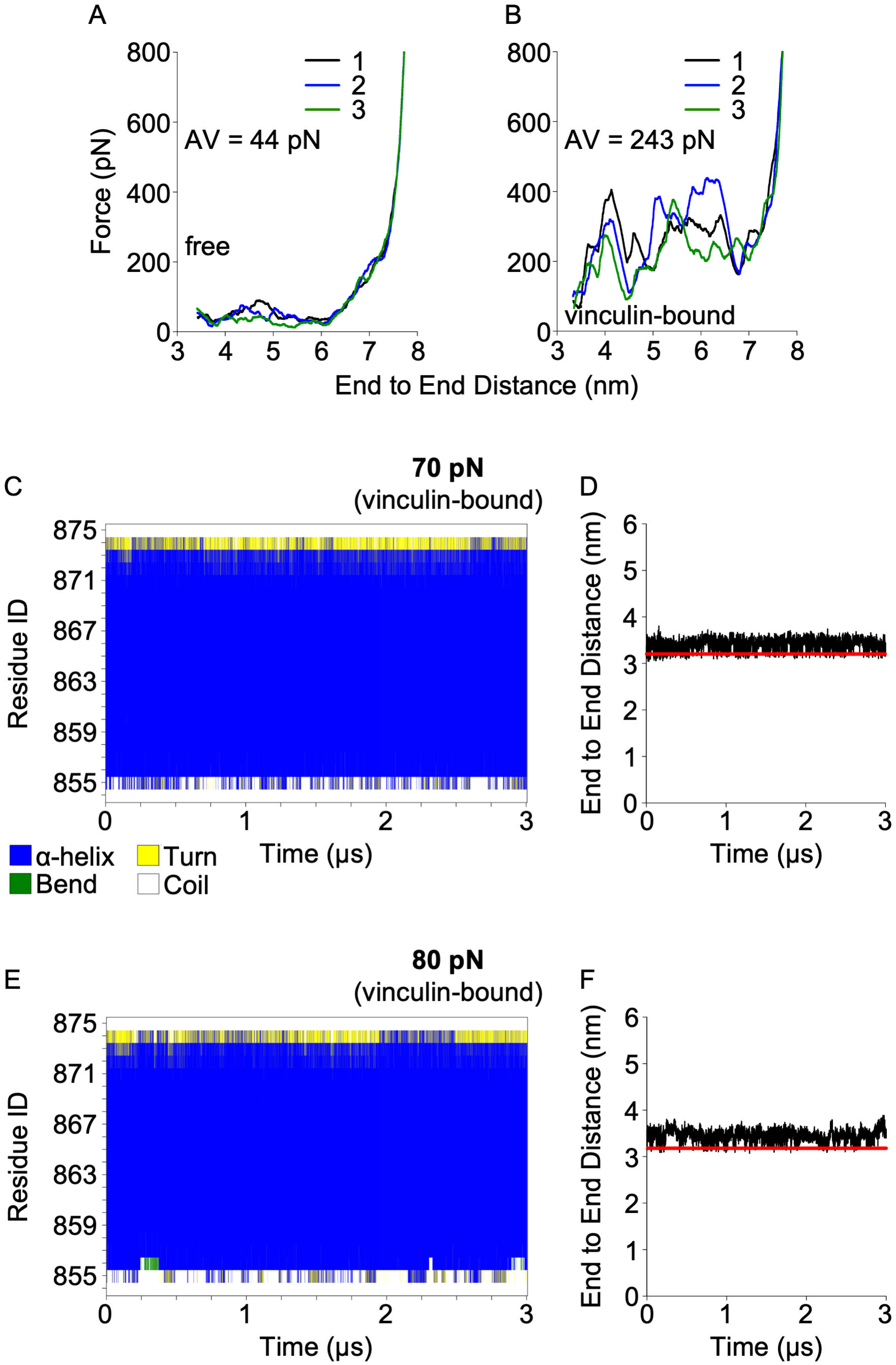

Supplement: S4 Fig — Unfolding force profiles for H12 R3 VBS in constant velocity SMD for (A) free and (B) complexed with vinculin. Secondary structure evolution and end-to-end distance for H12 R3 VBS in constant force SMD at (C,D) 70 pN and (E,F) 80 pN force regimes. (TIF) [file pcbi.1012341.s006.tif]

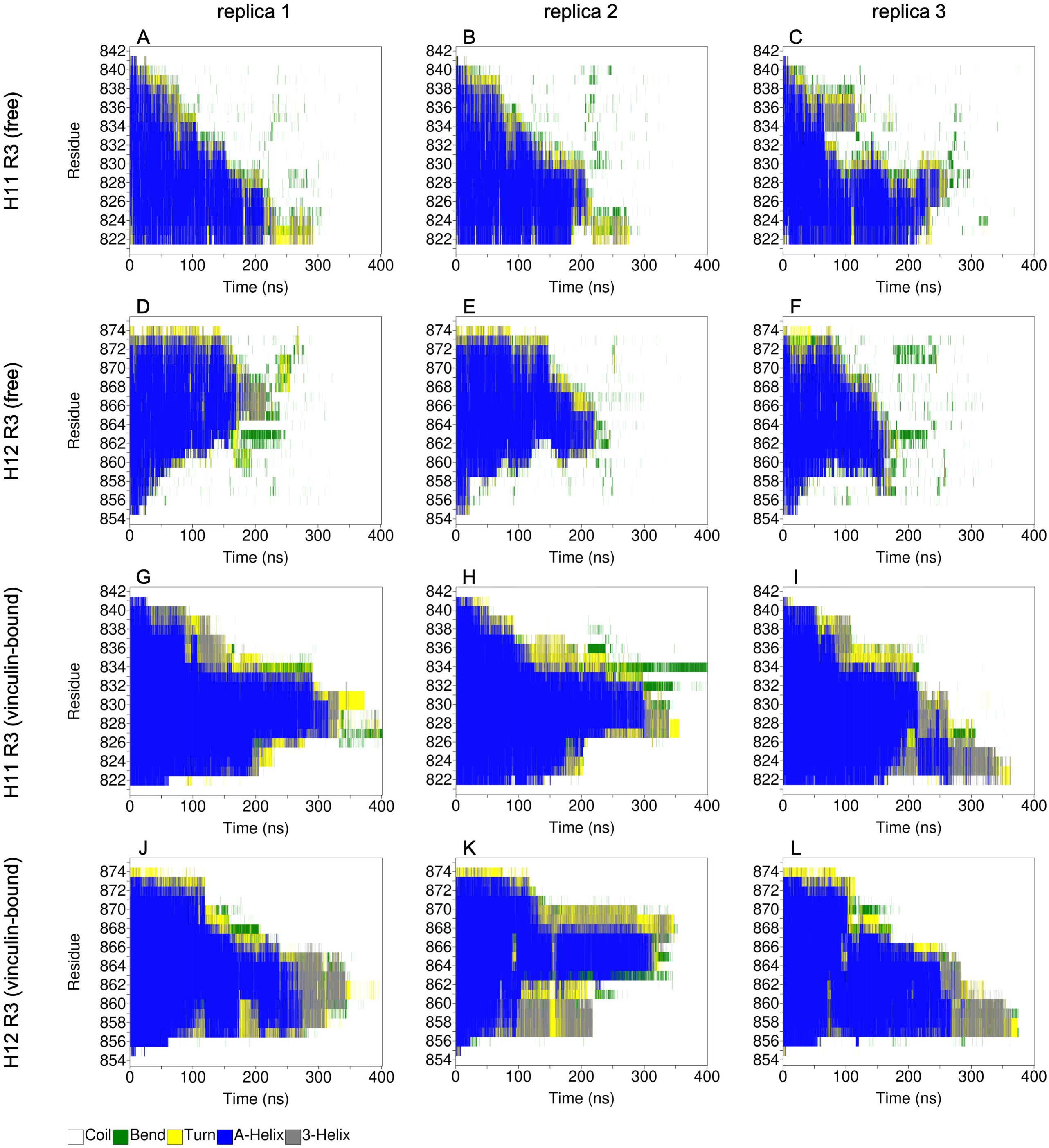

Supplement: S5 Fig — Secondary structure evolution plots for free and vinculin-bound (A-C, G-I) H11 R3 VBS, and (D-F, J-L) H12 R3 VBS. Three independent replicas were generated for each system. (TIF) [file pcbi.1012341.s007.tif]

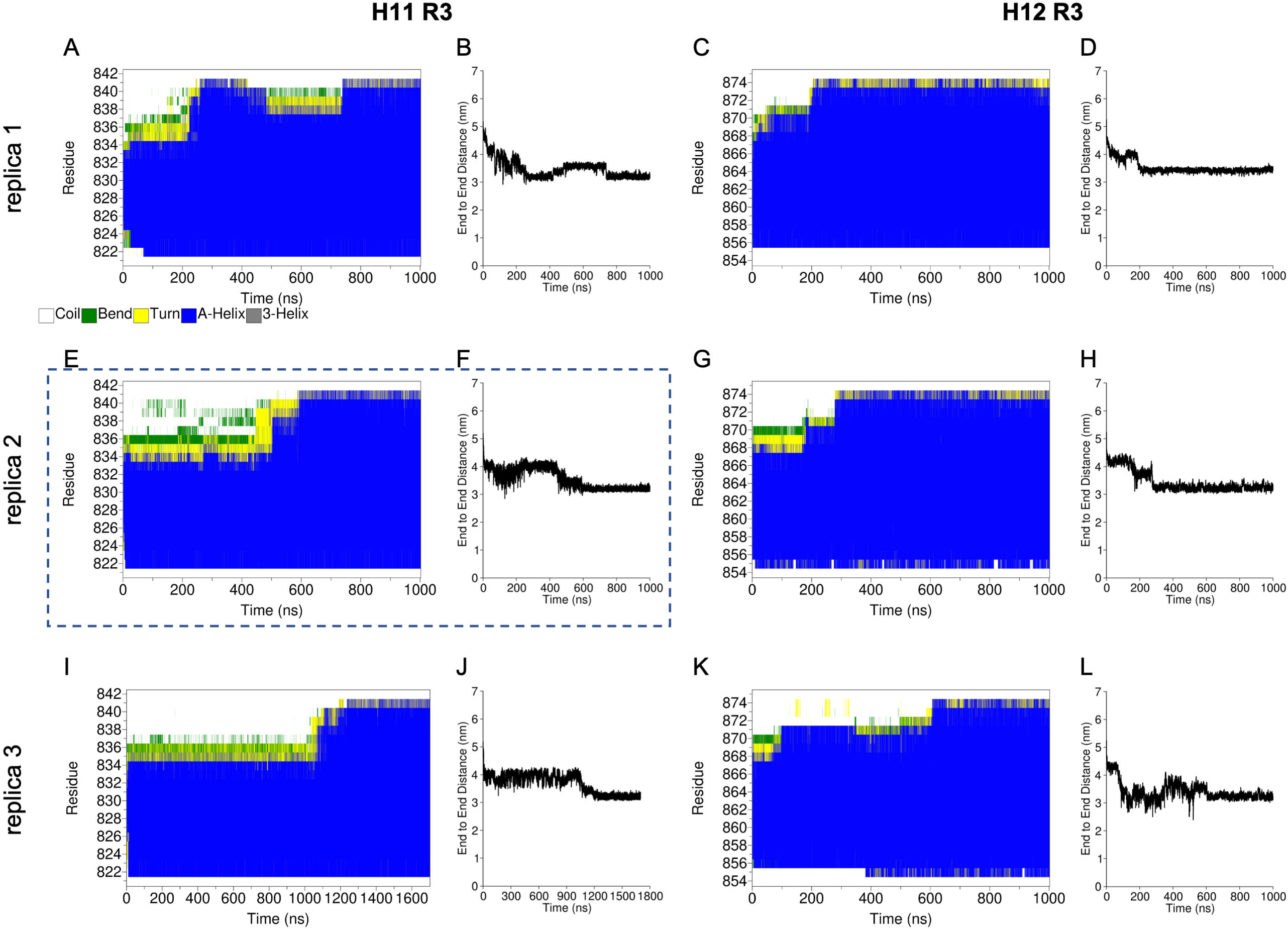

Supplement: S6 Fig — Secondary structure evolution and end-to-end distance for (A,B,E,F,I,J) H11 R3 VBS and (C,D,G,H,K,L) H12 R3 VBS in constant force SMD at 20 pN. Replica 2 (E,F) is shown in Fig 4C and 4D and is highlighted with dashed line box. (TIF) [file pcbi.1012341.s008.tif]

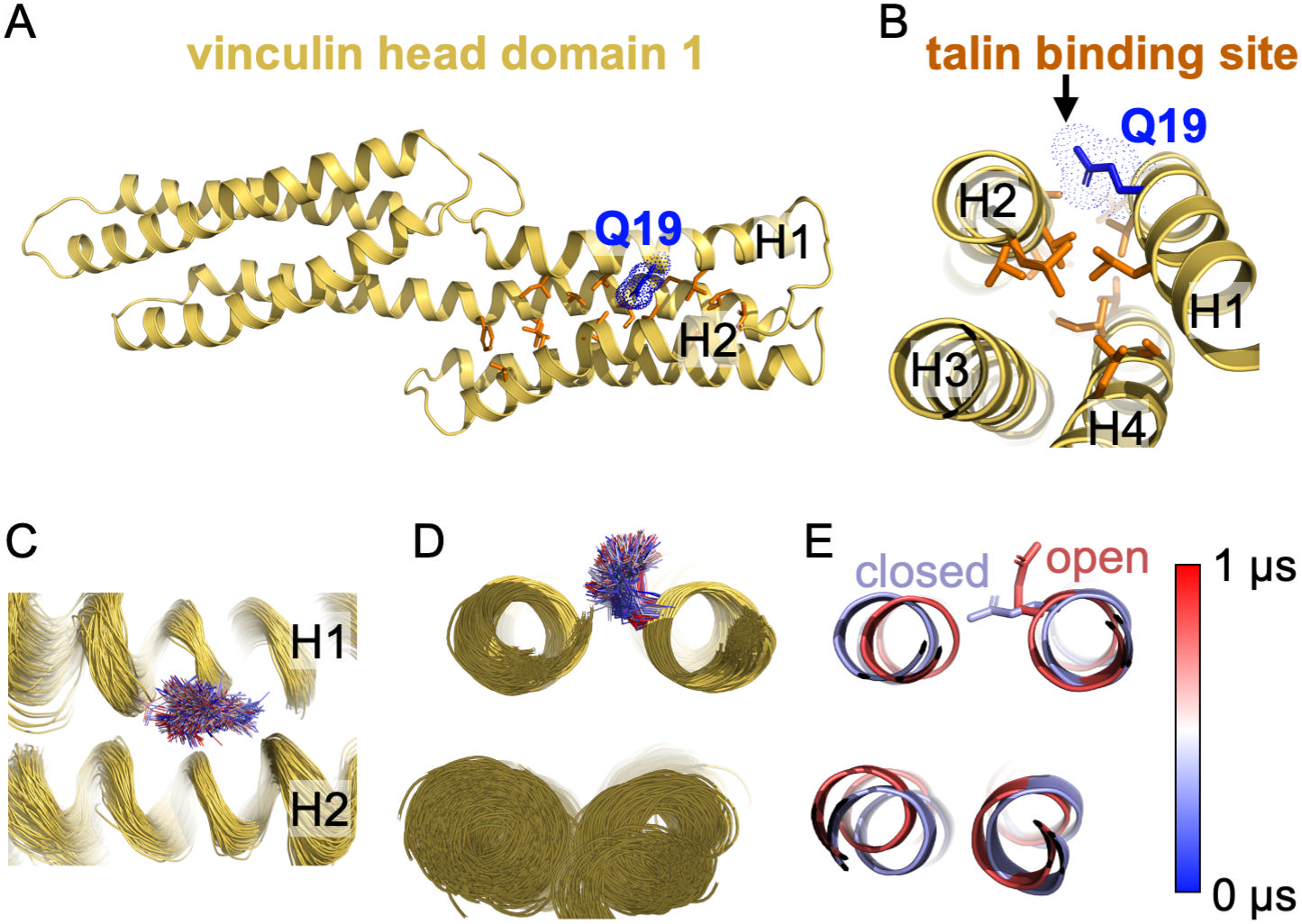

Supplement: S7 Fig — (A-B) Cartoon representation of VD1 tertiary structure, the Q19 side chain is shown in blue and the talin binding site in orange. (C-D) Superimposition of 1000 structure snapshots captured at every 1 ns from the 1 μs MD trajectory showing conformations of the Q19 sidechain. VD1 helices are shown as ribbons and Q19 sidechain as lines with the starting Q19 conformation in blue and the final confirmation in red. Superimposition performed using C-alpha atoms of Q19 and neighboring residues in the vinculin helices, H1 and H2. (E) Representative closed and open conformations of the Q19 sidechain shown as sticks. (TIF) [file pcbi.1012341.s009.tif]

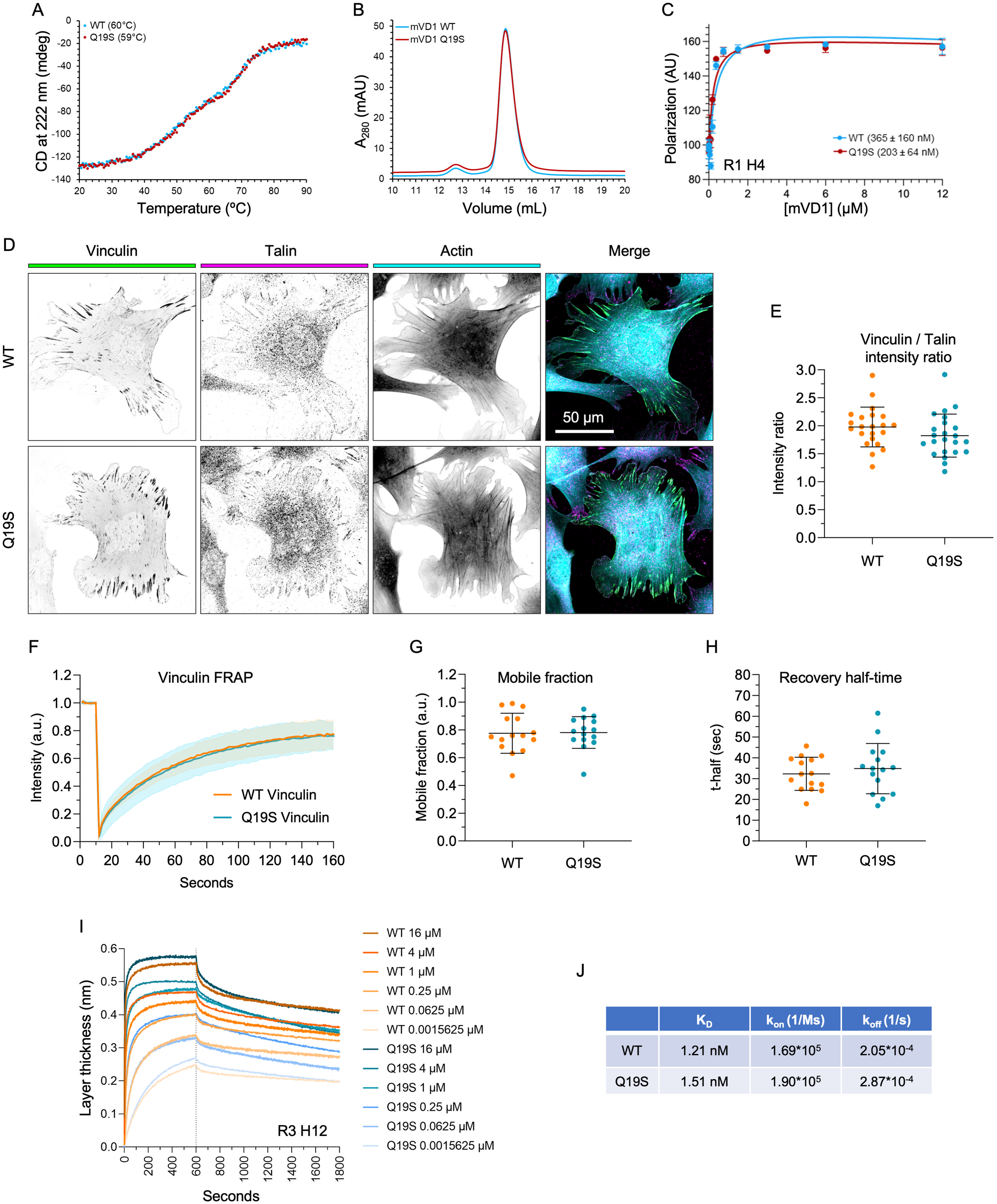

Supplement: S8 Fig — (A) Circular Dichroism (CD) thermal denaturation profiles of mVD1. Protein at 0.45 mg/mL was thermally denatured and the change in CD was monitored at 222 nm. The Tm of WT (blue) and Q19S (red) are shown in parentheses. (B) Oligomeric state analyzed by size exclusion chromatography. 100 μL of protein at 100 μM was loaded onto an S200 Increase G10/300 column and the absorbance was monitored at 280 nm. Both WT (blue) and Q19S (red) were monomeric in solution. Curves are offset by 1 mAU for clarity. (C) Fluorescence Polarization assay of WT (blue) and Q19S (red) protein binding a fluorescein labelled Tln1 H4 peptide. mVD1(Q19S) binds Tln1 H4 with a ~1.75-fold higher affinity than WT mVD1. The KD is shown in parentheses. (D) Representative images of vinculin-mEmerald or Q19S mutant localization in vinculin knock-out fibroblast cells. For individual channels, inverted fluorescence signal is shown. Merge image channel coloring is indicated by the colored bars above each individual channel. (E) Adhesion vinculin/talin intensity ratio in images of vinculin-mEmerald and anti-talin antibody staining. Each dot represents mean intensity ratio for a single cell. n = 22 and 23 cells for vinculin-mEmerald and Q19S mutant, respectively. (F) FRAP analysis of vinculin dynamics in vinculin knock-out fibroblast cells. Mean ± 1 SD. n = 15 cells for both vinculin variants, pooled from two independent experiments. (G-H) Mobile fraction and recovery half-time for FRAP data in F using a single-term curve fit. Mobile fraction of 1.0 indicates complete recovery of fluorescence signal. (I) Biosensor analysis of VD1 binding to biotinylated and immobilized on streptavidin-functionalized sensor talin H12 helix. WT vinculin head and Q19S mutant were incubated with H12-functionalized sensor tips for 600 sec using a 4-fold concentration series, followed by a dissociation phase for 1200 sec. (J) KD, kon and koff values determined using global fit to biosensor data shown in I. (TIF) [file pcbi.1012341.s010.tif]
